# Design of A-D-A-Type Organic Third-Order Nonlinear Optical Materials Based on Benzodithiophene: A DFT Study

**DOI:** 10.3390/nano12203700

**Published:** 2022-10-21

**Authors:** Pingping Gong, Lili An, Junfeng Tong, Xinpeng Liu, Zezhou Liang, Jianfeng Li

**Affiliations:** 1 Gansu Provincial Engineering Research Center for Organic Semiconductor Materials and Application Technology, School of Materials Science and Engineering, Lanzhou Jiaotong University, Lanzhou 730070, China; 2 Key Laboratory of Optoelectronic Technology and Intelligent Control of Ministry Education, Lanzhou Jiaotong University, Lanzhou 730070, China; 3 School of Chemical Engineering, Northwest Minzu University, Lanzhou 730030, China; 4 Key Laboratory of Physical Electronics and Devices of the Ministry of Education & Shaanxi Key Lab of Photonic Technique for Information, School of Electronic Science and Engineering, Faculty of Electronic and Information Engineering, Xi’an Jiaotong University, Xi’an 710049, China

**Keywords:** A-D-A-type structure, third-order nonlinear optical, density functional theory, second hyperpolarizability, conjugation length

## Abstract

The acceptor-donor-acceptor (A-D-A) type conjugated organic molecule has been widely applied in the organic optoelectronics field. A total of Nine compounds (**1**–**9**) were designed under the A-D-A framework, with the electron donor benzodithiophene as the core and dicyanomethylene as the acceptor moiety, modifying the benzodithiophene with the phenyl, naphthyl, and difluorinated phenyl groups. The conjugation length can be changed by introducing a thiophene π-conjugated bridge. The geometric structures, electronic structure, excited state properties, aromaticity, and the static- and frequency-dependent second hyperpolarizabilities were investigated by employing high-precision density functional theory (DFT) calculations with an aug-cc-pVDZ basis set. As a result, the three compounds with the longest conjugation length exhibit a smaller energy gap (*E*_gap_), larger UV-vis absorption coefficient, and response range, which are the three strongest third-order nonlinear optical (NLO) response properties in this work. This work systematically explored the connection between molecular structure and NLO response, which provides a rational design strategy for high-performance organic NLO materials.

## 1. Introduction

With the rapid development of nonlinear optics, nonlinear optical (NLO) materials have been widely applied in the field of fiber optics [1], data transformation [2], photonic lasers, and data storage in the area of wireless communication [3]. At present, the design and synthesis of high-performance NLO materials in organic and inorganic systems still meet major challenges [4]. In recent decades, many materials exhibited good optical nonlinearity, including organic materials [5,6,7,8], perovskite quantum dots [9], nanoclusters [10,11,12,13,14], organic-inorganic hybrid materials [1,15,16], etc. Among them, organic compounds that are easy to synthesize, with the advance of lower dielectric coefficients, have attracted wide attention [7].

Compared with traditional inorganic materials, organic chromophore compounds with excellent rapid natural response rates, a high absorption coefficient, tunable energy gaps, and molecular structures are easily adjustable by molecular designing, which is considered promising for NLO materials [17]. Organic-photovoltaic (OPV) materials include electronic donors and acceptors that, based on the donor-acceptor-type (D-A) and donor-π-acceptor-type (D-π-A) molecular structures, have been widely applied in OPV devices and achieved exciting power conversion efficiency. Because of the highly π-conjugated D-A/D-π-A molecular structure, the intramolecular charge transfer (ICT) would be easier and greater for obtaining efficient NLO characteristics [7]. The properties of substituent groups and the conjugated length of the molecules can significantly influence NLO characteristics.

**Figure 1:**
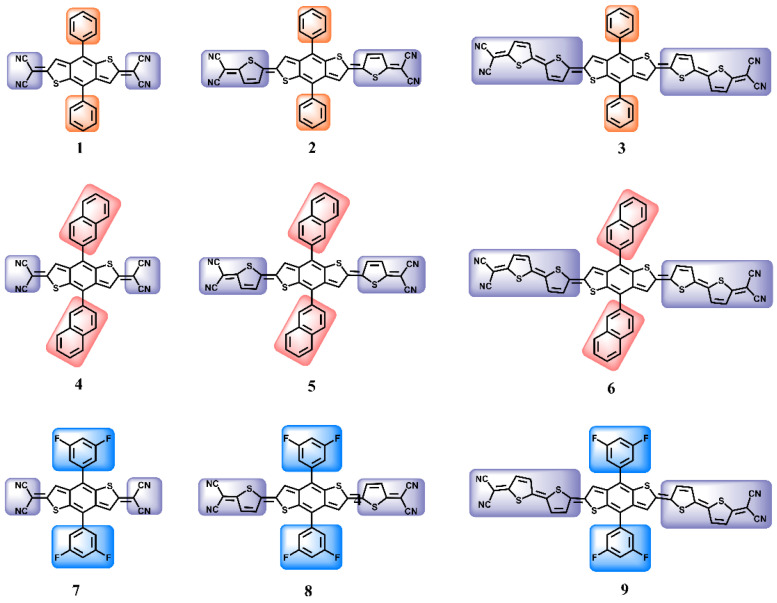
Chemical structures of the nine compounds (**1**–**9**).

In this work, benzodithiophene was selected as the core, which is generally a key structure of the donor unit in D-A/D-π-A polymer electronic donors in OPV [18,19,20,21,22,23,24]. For the NLO materials, the centrosymmetry is a basic structural feature, and the molecular structure of the D-A-D type has symmetry itself. For organic molecules, because the D-A-D molecular structure has a significant ICT effect, it is easy to achieve better photo response characteristics, and the ICT of molecules can be adjusted by changing the electronic structure of the side chains and the conjugate length of the molecules so that nine molecules were designed with a terminal dicyanomethylene acceptor as the end-group, and thiophene as a conjugate extension, meaning that the electron-deficient terminal groups could be conducive to enhancing the molecule’s absorption coefficient. Among the nine compounds, as displayed in Figure 1, compounds **1**–**3**, **4**–**6**, and **7**–**9** were designed by extending the molecule’s conjugated length by increasing the thiophene rings. The compounds **1**–**3**, **4**–**6**, and **7**–**9** modified the benzodithiophene with phenyl, naphthyl, and difluorinated phenyl groups, respectively. Based on this modification strategy, the influence of the conjugated length of the molecule and the molecular electronic structure changes on linear optical and third-order NLO properties were deeply explored for compounds **1**–**9**. The molecular electronic structure, energy gap, second hyperpolarizability (*γ*), UV−vis spectra, excited hole-electron character, and anisotropy of the induced current density (AICD) are comprehensively investigated by density functional theory (DFT) calculations. The calculated results indicate that compounds **3**, **6**, and **9** exhibited marvelous third-order NLO properties. With the compound’s conjugation length increased, the *E*_gap_ is smaller, and the UV-vis response range is wider, with the linear response intensity and the third-order NLO response being stronger. On account of this, this NLO material design strategy will help to develop new high-performance organic NLO materials.

## 2. Computational Methods

All the DFT calculations were carried out using the Gaussian 16 package [25], and the Visual Molecular Dynamics (VMD) software was employed to visualize the calculated results [26]. All the geometries were optimized under the B3LYP-D3(BJ)/def2SVP level [27,28]. After geometry optimization, we further confirmed no imaginary frequency by vibrational frequency calculation. The excited states of the nine geometries were calculated by the time-dependent density functional theory (TD-DFT) using the CAM-B3LYP method with a def2SVP basis set [29]. In order to obtain proficiency and accuracy, static and frequency-dependent third-order NLO hyperpolarizabilities (*γ*) were calculated using the CAM-B3LYP method by using the high accuracy aug-cc-pVDZ basis set [30,31,32,33,34,35,36,37,38,39]. The molecule’s electronic structure, hyperpolarizability, excited state properties, and aromaticity were analyzed by Multiwfn 3.8(dev) code [40].

The second hyperpolarizability density $\rho_{xxx}^{\left(3\right)}$ of nine molecules is contrasted between the dipole moment *μ*(*F*) after Taylor expansion and electron density, as follows [7,11].

(1) $$E\left(F\right)=E\left(0\right)-\mu_{0}F-\frac{1}{2}\alpha F^{2}-\frac{1}{6}\beta F^{3}-\frac{1}{24}\beta F^{4}-\ldots$$

(2) $$\mu \left(F\right)=-\frac{\partial E}{\partial F}=\mu_{0}+\alpha F+\frac{1}{2}\beta F^{2}+\frac{1}{2}\beta F^{3}+\ldots$$

(3) $$\rho \left(r,F\right)=\rho^{\left(0\right)}\left(r\right)+\rho^{\left(1\right)}\left(r\right)F+\frac{1}{2}\rho^{\left(2\right)}\left(r\right)F^{2}+\frac{1}{6}\rho^{\left(3\right)}\left(r\right)F^{3}+\ldots$$

where *μ*_0_ is the permanent dipole moment and *F* is the uniform external electric field.

(4) $$\gamma_{xxx}=\int -\rho_{xxx}^{\left(3\right)}\left(r\right)xdx$$

(5) $$\rho_{xxx}^{\left(3\right)}=\frac{\rho \left(2F^{x}\right)-2\rho \left(F^{x}\right)+2\rho \left(-F^{x}\right)-\rho \left(-2F^{x}\right)}{2{\left(F^{x}\right)}^{3}}$$

The *i* components of *γ* are defined as

(6) $$\gamma_{i}=\left(\frac{1}{15}\right)\underset{j}{\sum }\left(\gamma_{ijji}+\gamma_{ijij}+\gamma_{iijj}\right)i,j=\left\{x,y,z\right\}$$

The total magnitude of *γ* is measured as

(7) $$\gamma_{tot}=\sqrt{\gamma_{x}^{2}+\gamma_{y}^{2}+\gamma_{z}^{2}}$$

## 3. Results and Discussion

**Figure 2:**
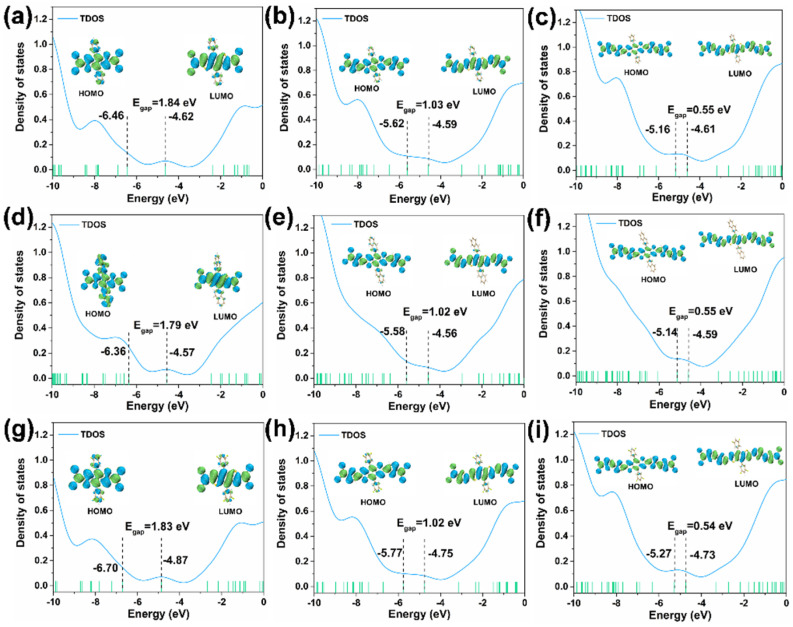
DOS curves of the nine compounds. (**a**–**i**) are compounds (**1**–**9**).

All the geometries were optimized in the gas phase under the B3LYP-D3(BJ)/def2SVP level. As shown in Figure 2, Figures S1 and S2, all the molecules are rigid and planar along the main skeleton. The HOMOs (highest occupied molecular orbitals), LUMOs (lowest unoccupied molecular orbitals), and the energy gaps (*E*_gap_) of the nine molecules are calculated as displayed in Figure 2. The HOMO energy of compound **1** was −6.46 eV, with a LUMO energy of −4.62 eV and an *E*_gap_ of 1.84 eV. Similarly, the HOMO and LUMO energy of compound **2** were −5.62 eV and −4.59 eV, respectively, with an *E*_gap_ of 1.03 eV. The remaining compounds **3**, **4**, **5**, **6**, **7**, **8**, and **9** had a HOMO energy of −5.16, −6.36, −5.58, −5.14, −6.70, −5.77, and −5.27 eV, respectively. While their LUMO energy was found to be −4.61, −4.57, −4.56, −4.59, −4.87, −4.75, and −4.73 eV, respectively, with an *E*_gap_ of 0.55, 1.79, 1.02, 0.55, 1.83, 1.02, and 0.54 eV, respectively. The order of *E*_gap_ for the compounds was: compound **1** < compound **2** < compound **3**, compound **4** < compound **5** < compound **6**, compound **7** < compound **8** < compound **9**. It is clearly shown that with an increase in the molecule’s conjugated length, the *E*_gap_ decreased. The TDOS (total density of states) plots were calculated to show the population evaluation of each orbital, which reflects the number of molecular orbitals (MOs) in the unit energy interval at the corresponding energy.

**Figure 3:**
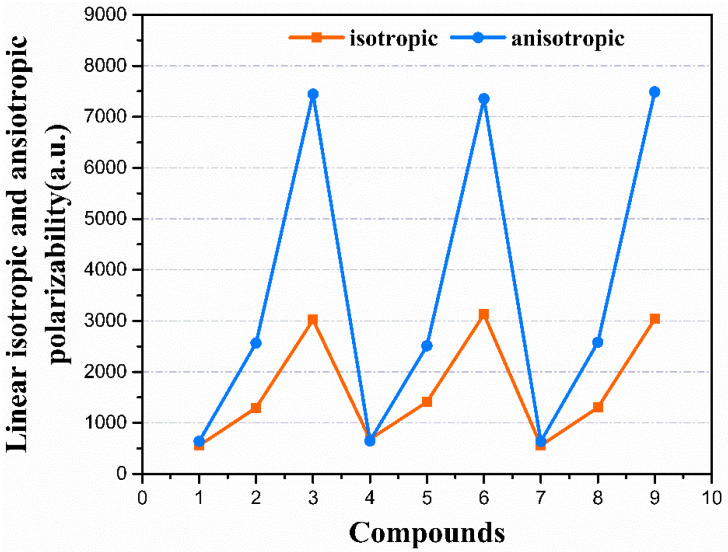
The linear isotropic (α_iso_) and anisotropic (α_aniso_) polarizability.

There is an important connection between the molecule charge distribution and linear molecular polarizability (α) [41]. The molecular polarizability can be influenced by the applied external electric field. The linear isotropic (α_iso_) and anisotropic (α_aniso_) polarizabilities, as well as the individual tensor components of all the compounds, were calculated at the CAM-B3LYP/aug-cc-PVDZ level, as given in Table 1. The α_xx_ shows larger values among the tensor components, which suggests the polarizability mainly occurred along the *x*-axis. Compounds **3**, **6**, and **9** show larger polarizability values of 7979.27, 8018.49, and 8023.75 a.u., respectively. They are much larger than the other remaining compounds. The compounds exhibited a trend of increasing linear polarizabilities, which corresponds well with the trend of increasing the conjugated length of the molecule, as **1** < **2** < **3**, **4** < **5** < **6**, **7** < **8** < **9**. As displayed in Figure 3, the α_iso_ and α_aniso_ increased with the increments in π-conjugation length. For all the compounds, compound **1** exhibits the smallest value of α_iso_ and α_aniso_, which were 556.12 and 638.61 a.u., respectively. However, compound **9** exhibited the largest α_iso_ and α_aniso_ of 3035.44 and 7489.37 a.u., respectively. The large difference between the α_iso_ and α_aniso_ values for all the compounds suggests that the direction of the response of these materials to external electric fields is significant selectively.

**Figure 4:**
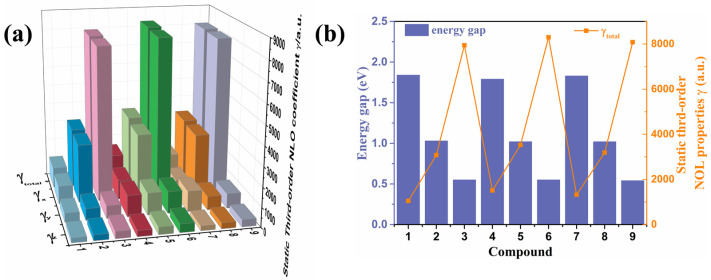
(**a**) Static third-order NLO coefficients (*γ*) of the compounds (**1**–**9**); (**b**) the relationship between the static third-order NLO polarizabilities and *E*_gap_.

The second-order hyperpolarizability (*γ*) is considered to be an important index to measure nonlinear optical coefficients. The *γ* values were calculated under the CAM-B3LYP/aug-cc-PVDZ level, which is shown in Figure 4a and is summarized in Table S1. The results of static third-order NLO coefficients (*γ*_tot_) of these nine compounds show that when the backbone has the same conjugate length, the *γ*_tot_ of those molecules with difluorinated phenyl groups is stronger than that of phenyl- and naphthyl-modified side groups. As shown in Figure 4b, the results suggest that, for those compounds in which the *E*_gap_ is smaller, the polarizability is larger for their smaller excitation energy. To further investigate the NLO properties of the nine compounds, the second hyperpolarizabilities *γ* (−2ω; ω, ω, 0) were calculated, as summarized in Table 2. The sequence of the *γ* (−2ω; ω, ω, 0) values were *γ* (∞ nm) < *γ* (1064 nm) < *γ* (532 nm) for compounds **1**, **2**, **4**, **5**, **7**, and **8**, and for compounds **3**, **6** and **9**, the *γ* (−2ω; ω, ω, 0) values were *γ* (532 nm) < *γ* (∞ nm) < *γ* (1064 nm). Which are higher than the reported bis(dicyanomethylene) end-functionalized quinoidal oligothiophenes [7].

**Figure 5:**
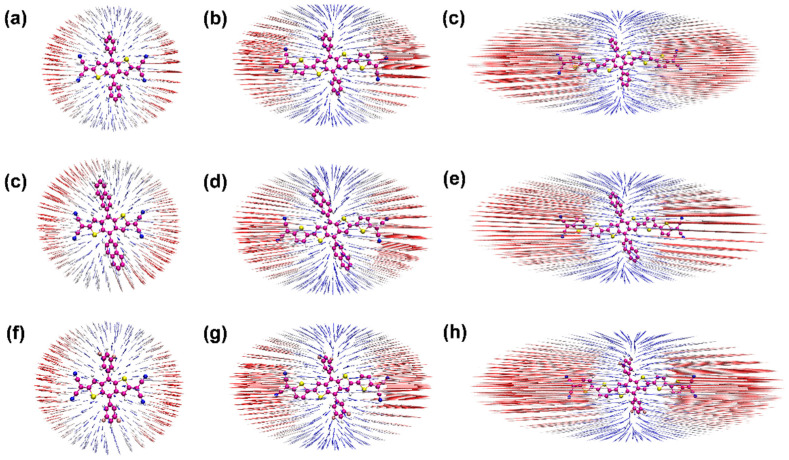
(**a**–**h**) the anisotropy of the hyperpolarizability of the nine compounds (**1**–**9**); the blue and red represent arrow difference in length.

**Figure 6:**
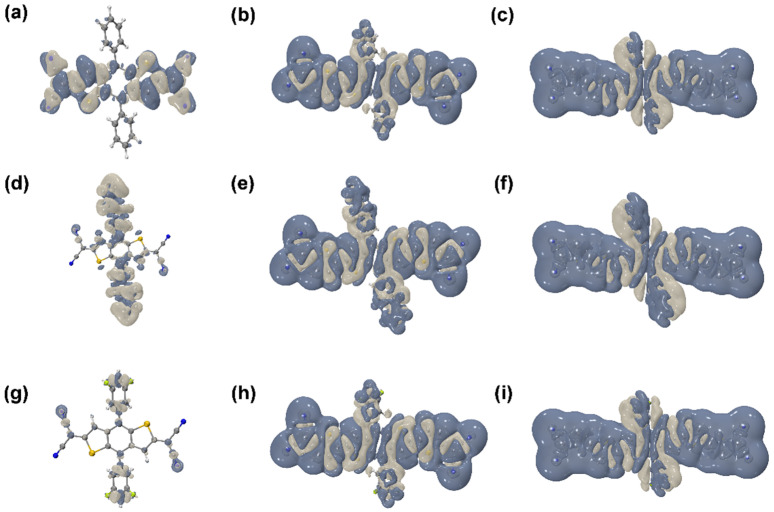
(**a**–**i**) are the isosurfaces of $-{Xp}_{xxx}^{\left(3\right)}(r)$ (isovalue = 1100) of the nine compounds (**1**–**9**).

The anisotropy of the third-order NLO polarizabilities can be visualized directly, perceived through the unit sphere representation [11,42,43], as shown in Figure 5. The change of the molecular dipole moment can be measured based on the unit sphere representation due to the length and direction of the arrows, representing the strength and direction of the dipole moment, which can show the direction and intensity of the *γ*. Furthermore, the hyperpolarizability density was calculated using the numerical differentiation method, and the contribution of hyperpolarizability in different regions can be clearly presented. Based on the calculated hyperpolarizability density results, as shown in Figure 6, the gray and blue-black regions denote positive and negative spatial contributions, with superposition effects for all the compounds, and the hyperpolarizability density can clearly show the magnitude of hyperpolarizability and the main contribution regions. All the distributions of $-{Xp}_{xxx}^{\left(3\right)}(r)$ are centrosymmetric for the nine centrosymmetric molecules [42,43]. Moreover, compared with Figure 5 and Figure 6, it can be seen that the length of the π-conjugate has a significant positive effect on the third-order NLO response.

**Figure 7:**
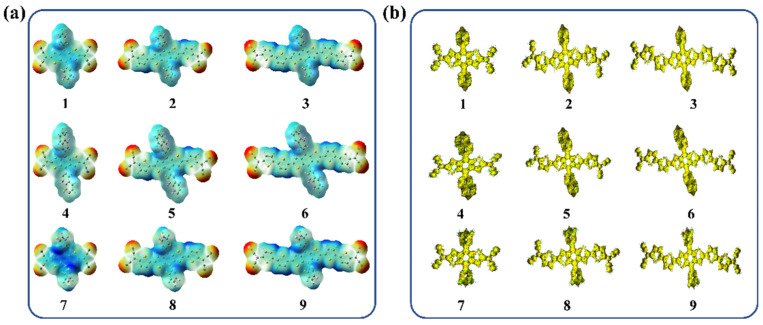
(**a**) The molecular surface electrostatic potentials of all the compounds (**1**–**9**), red and blue, represent the negative and positive potential, respectively; (**b**) AICD plots (isovalue: 0.03 a.u.) of the compounds (**1**–**9**).

The molecular surface electrostatic potentials of the compounds were further calculated to understand the characteristics of charge distribution on the molecule surface. As shown in Figure 7a, the red and blue colors represent the relative negative and positive potential surfaces, respectively, and the terminal dicyanomethylene groups show a maximum negative potential. The maximum positive potential region appears on the central benzodithiophene core or π-bridge (thiophene moiety). In order to further confirm the molecule aromaticity, the anisotropy of the induced current density (AICD) plot of the compounds was further calculated, as shown in Figure 7b. The results indicated that all the compounds would generate ring current under a magnetic field, π-electrons delocalized within the whole molecule, meaning they are aromatic.

**Figure 8:**
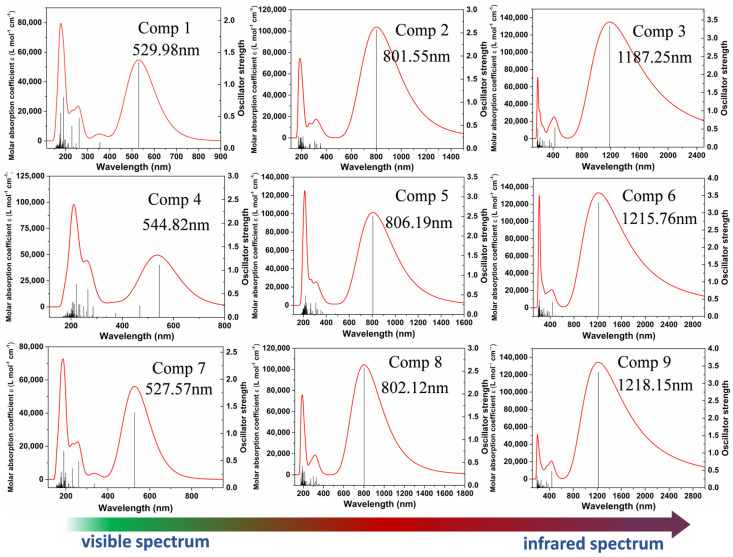
The UV−vis spectra of the nine compounds.

As displayed in Figure 8, the absorption spectra of the compounds were obtained by TDDFT calculations at the CAM-B3LYP/def2SVP level. We first obtained the excited state energy and oscillator strength, and the excited state energy was converted into nm units. They were then expanded into curves and added together, and the spectra were broadened by a Gaussian function with a full width at half maximum (FWHM) of 0.6667 eV. The transition energy *E* (eV), oscillator strength (*f*), maximum absorption wavelength λ_max_ (nm), and molecular orbit transition contributions of the investigated compounds (**1**–**9**) are presented in Table 3. The calculated λ_max_ of the compounds (**1**–**9**) is attributed to the electron excited from the ground state to the first excited state. The simulated compounds absorption spectra ranged from visible light and extended into the infrared region. The simulated compounds spectra scale shows a redshift tendency, and the oscillator strength is simultaneously enhanced with the increasing π-conjugate length of the molecule. Meanwhile, in order to further confirm the reliability of the simulated spectra, all the molecules have been simulated using the wB97XD method to calculate the UV-Vis spectra, which was in good agreement with CAM-B3LYP methods, as shown in Figure S3.

**Figure 9:**
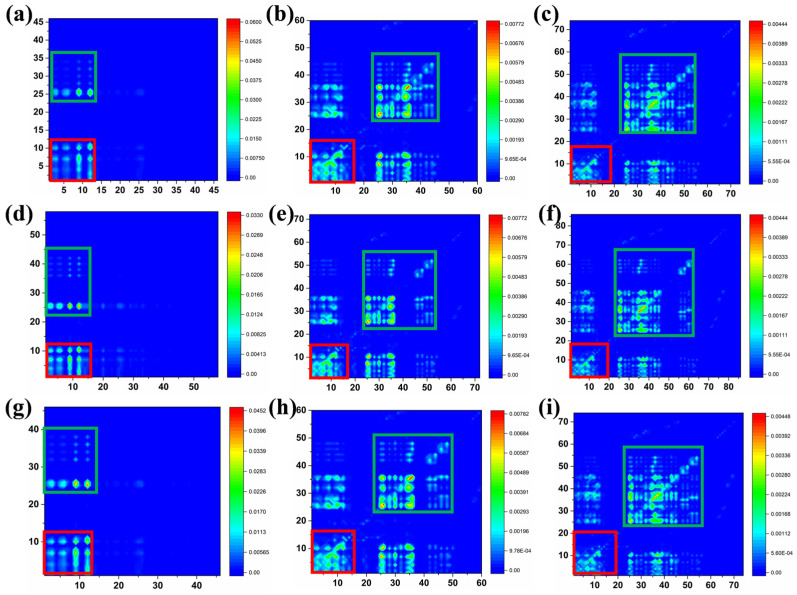
(**a**–**i**) TDM plots of the compounds (**1**–**9**).

In order to deeply understand the charge transfer characteristics of the molecules in their excited states, we calculated the transition density matrix (TDM) plots, as shown in Figure 9. The TDM plots can be more intuitive in reflecting the information of the ICT as well as the specific contributions of the electrons, holes, and electron-hole overlaps. Here, the red frame region represents the central benzodithiophene moiety, and the green frame region represents the thiophene, phenyl, naphthyl, and difluorinated phenyl or C(CN)_2_ groups. Based on the calculated TDM results, all the molecules exhibited significant ICT behavior. Except for compounds **1**, **3**, and **5**, the electron-hole pairs of other compounds were mainly localized along with the diagonal element. The results suggest the electron transition involves the whole molecule, which is beneficial for intramolecular charge transfer. Compounds **1**, **3**, and **5** with the structure of the benzodithiophene are substituted with the phenyl, naphthyl, and difluorinated phenyl groups, and the benzodithiophene is directly combined with the dicyanomethylene end-group. For the small, conjugated distance, for compounds **1**, **3**, and **5**, the twist structure between benzodithiophene and the side substituents (phenyl, naphthyl, and difluorinated phenyl groups) had lost the delocalized electric to a great degree so that the structure-property of compounds **1**, **3**, and **5** further enhanced the electron-hole transition coherence and did not benefit from ICT, as displayed in Figure 9a,d,g. Among all the compounds, compounds **3**, **6**, and **9** show the most remarkable ICT effects, followed by the largest molecule conjugation length and smaller *E*_gap_. Moreover, the off-diagonal part of the TDM also exhibited the probability of electron transformation, which contributed to the molecule’s charge transfer. 

## 4. Conclusions

In this contribution, nine molecules based on benzodithiophene as the core- and dicyanomethylene as the end-group were designed, with their molecule conjugation length changed by increasing the number of thiophene moieties. The molecular electronic structure, TDOS, molecular surface electrostatic potential, anisotropy of the AICD, UV-vis absorption spectra, and TDMs were calculated by DFT and TDDFT to deeply investigate the compounds. The calculated results demonstrate that compounds **3**, **6**, and **9**, with long conjugated lengths, exhibited high third-order NLO responses for their smallest *E*_gap_ and large UV-vis absorption coefficients. The TDM results also proved that an increase in conjugated length could promote ICT. Therefore, the enhancement of nonlinear optical response properties is mainly caused by an increase in molecule conjugated length. At the same time, the electronic structure of the side chain groups also affects NLO, and modifying those groups with strong electronegativity will enhance ICT and further enhance the NLO. Therefore, to design high-performance NLO molecular materials with an A-D-A structure, researchers should consider increasing the conjugated length of the molecule and introducing electron-withdrawing groups to the donor or acceptor units. The object of our research was to abstract molecules into isolated systems, which has a certain reference significance for the performance of the actual molecules. This work provides a strategy to design and synthesize new organic third-order NLO materials with highly responsive characteristics. 

## Figures and Tables

**Table 1:** Calculated α (a.u.) and the individual components.

α	Comp 1 (a.u.)	Comp 2 (a.u.)	Comp 3 (a.u.)	Comp 4 (a.u.)	Comp 5 (a.u.)	Comp 6 (a.u.)	Comp 7 (a.u.)	Comp 8 (a.u.)	Comp 9 (a.u.)
α_xx_	952.08	2996.13	7979.27	810.35	3028.70	8018.49	575.88	3007.86	8023.75
α_xy_	−34.43	5.86	−69.60	180.20	292.75	190.93	186.85	92.34	15.61
α_yy_	489.14	598.37	726.34	921.301	834.517	928.69	865.15	601.55	726.26
α_xz_	0.000020	−0.0068	0.02210	−0.000030	0.00402	0.036	−0.000020	−0.0094	0.023
α_yz_	−0.000030	−0.0059	−0.00531	−0.000020	0.00715	0.010	0.000010	−0.017	−0.0052
α_zz_	227.12	290.63	354.587	308.309	371.897	437.18	231.75	293.634	356.31
α_iso_	556.12	1295.05	3020.06	679.986	1411.7	3128.12	557.59	1301.02	3035.40
α_aniso_	638.61	2565.53	7446.75	646.123	2510.11	7355.33	637.49	2579.08	7489.40

**Table 2:** Calculated static and dynamic second hyperpolarizability (*γ*).

Incident Light	Second Hyperpolarizability (*γ*)								
	Comp 1 (a.u.)	Comp 2 (a.u.)	Comp 3 (a.u.)	Comp 4 (a.u.)	Comp 5 (a.u.)	Comp 6 (a.u.)	Comp 7 (a.u.)	Comp 8 (a.u.)	Comp 9 (a.u.)
∞ (static)	1.94 × 10^5^	1.85 × 10^6^	4.18 × 10^7^	3.29 × 10^5^	2.40 × 10^6^	4.22 × 10^7^	2.45 × 10^5^	1.84 × 10^6^	4.02 × 10^7^
532 nm	1.18 × 10^10^	6.70 × 10^7^	1.70 × 10^7^	1.02 × 10^9^	9.42 × 10^7^	2.59 × 10^7^	1.66 × 10^9^	1.68 × 10^8^	2.77 × 10^7^
1064 nm	3.01 × 10^7^	3.66 × 10^7^	5.81 × 10^8^	1.01 × 10^7^	3.78 × 10^7^	2.73 × 10^9^	3.13 × 10^7^	3.83 × 10^7^	1.41 × 10^9^

**Table 3:** Excitation energy (*E*), oscillator strengths (*f*), wavelengths (λ_max_), and MO contributions of the compounds.

Compound	λ_max_ (nm)	*E*(eV)	*f*	MO Contributions ^a^
1	529.98	2.34	1.35	H → L (98.07%)
2	801.55	1.54	2.56	H → L (97.40%)
3	1187.25	1.04	3.34	H → L (98.03%)
4	544.82	2.27	1.12	H → L (98.00%)
5	806.19	1.54	2.51	H → L (97.50%)
6	1215.76	1.02	3.29	H → L (97.90%)
7	527.57	2.35	1.39	H → L (98.18%)
8	802.12	1.55	2.58	H → L (97.50%)
9	1218.15	1.01	3.32	H → L (98.00%)

^a^ H = HOMO, L = LUMO.

## Data Availability

Not applicable.

## Supplementary Materials

The following supporting information can be downloaded at: https://www.mdpi.com/article/10.3390/nano12203700/s1; Figure S1: The front view of the optimized molecules; Figure S2: The side view of the optimized molecules; Table S1: Static third-order NLO coefficients (*γ*) of the nine molecules; Figure S3: The UV-vis spectra of the nine compounds calculated under the wB97XD/def2-SVP level.
