# Supplementary material for: Design of A-D-A-Type Organic Third-Order Nonlinear Optical Materials Based on Benzodithiophene: A DFT Study
Source: Nanomaterials (Basel). 2022 Oct 21;12(20):3700. doi: 10.3390/nano12203700 (PMC9611063; doi:10.3390/nano12203700)
Supplement: Supplementary file 1 [file nanomaterials-12-03700-s001.zip › nanomaterials-1943613-supplementary.pdf]

# Design of A-D-A-Type Organic Third-Order Nonlinear Optical Materials Based on Benzodithiophene: A DFT Study

**Pingping Gong [1], Lili An [2,3], Junfeng Tong [1,*], Xinpeng Liu [1], Zezhou Liang [4,*] and Jianfeng Li [1]**

1 Gansu Provincial Engineering Research Center for Organic Semiconductor Materials and Application Technology, School of Materials Science and Engineering, Lanzhou Jiaotong University, Lanzhou 730070, China
2 Key Laboratory of Optoelectronic Technology and Intelligent Control of Ministry Education, Lanzhou Jiaotong University, Lanzhou 730070, China
3 School of Chemical Engineering, Northwest Minzu University, Lanzhou 730030, China
4 Key Laboratory of Physical Electronics and Devices of the Ministry of Education & Shaanxi Key Lab of Photonic Technique for Information, School of Electronic Science and Engineering, Faculty of Electronic and Information Engineering, Xi’an Jiaotong University, Xi’an 710049, China
* Correspondence: tongjunfeng139@163.com (J.T.); zezhouliang@foxmail.com (Z.L.)

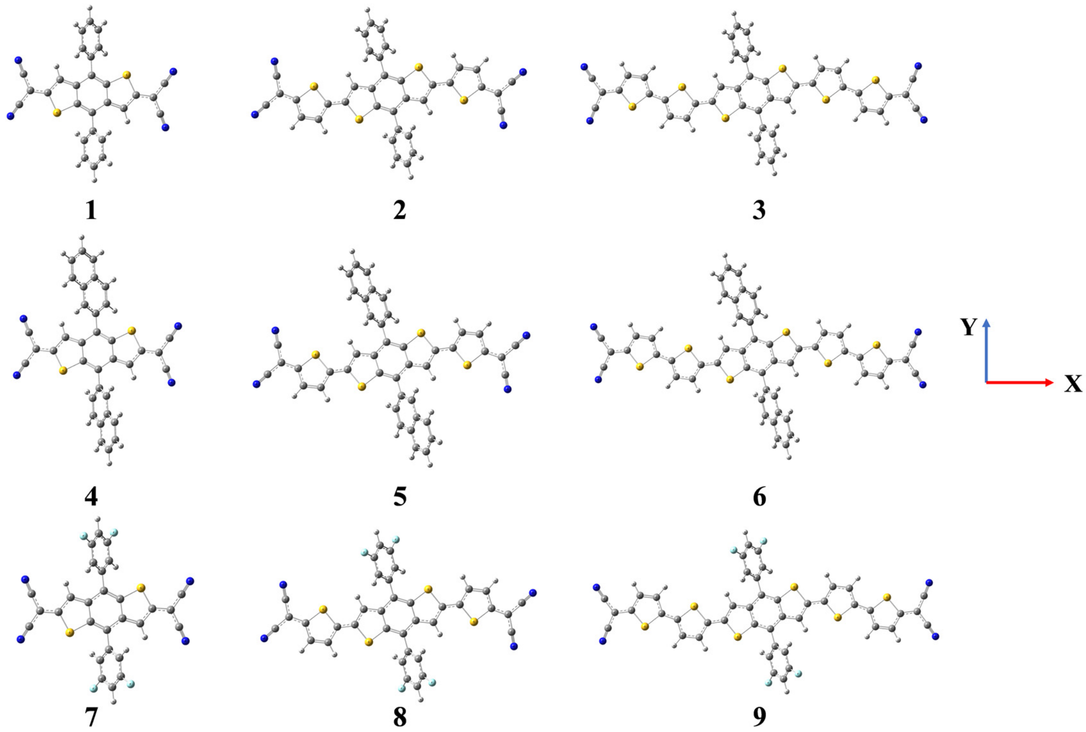


Figure S1. The front view of the optimized molecules.

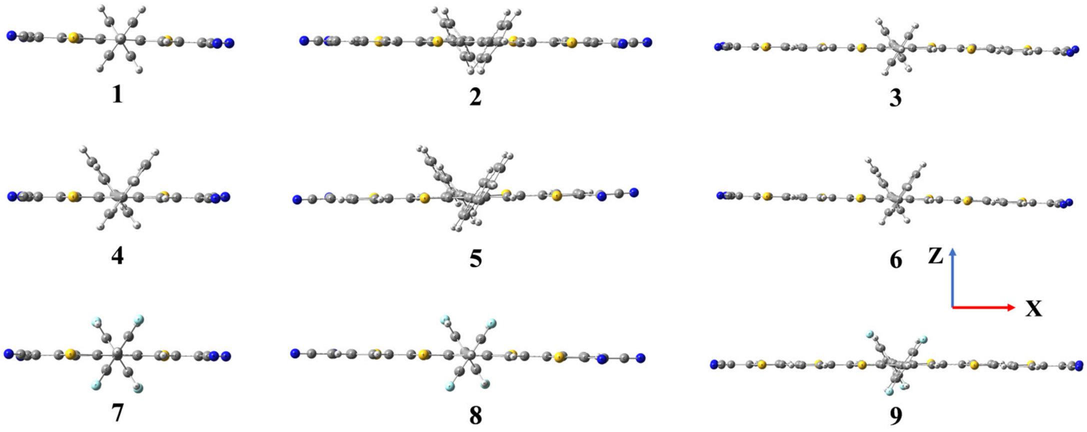


Figure S2. The side view of the optimized molecules.

**Table S1**. Static third-order NLO coefficients (γ) of the nine molecules.

| molecules | $\gamma_{total}$ | $\gamma_x$ | $\gamma_y$ | $\gamma_z$ |
|---|---|---|---|---|
| 1 | 1049.01004 | 917.64652 | 454.71147 | 227.12199 |
| 2 | 3075.94347 | 3001.97982 | 604.22474 | 290.61633 |
| 3 | 7944.82987 | 7909.6953 | 656.73289 | 354.60379 |
| 4 | 1513.12745 | 990.54997 | 1101.50398 | 308.30895 |
| 5 | 3527.2009 | 3321.45702 | 1127.27715 | 371.90817 |
| 6 | 8296.9846 | 8209.45886 | 1119.63029 | 437.22615 |
| 7 | 1319.91511 | 762.73198 | 1052.00201 | 231.749 |
| 8 | 3190.42442 | 3100.18606 | 693.86384 | 293.6109 |
| 9 | 8081.39683 | 8039.38096 | 741.86364 | 356.32401 |

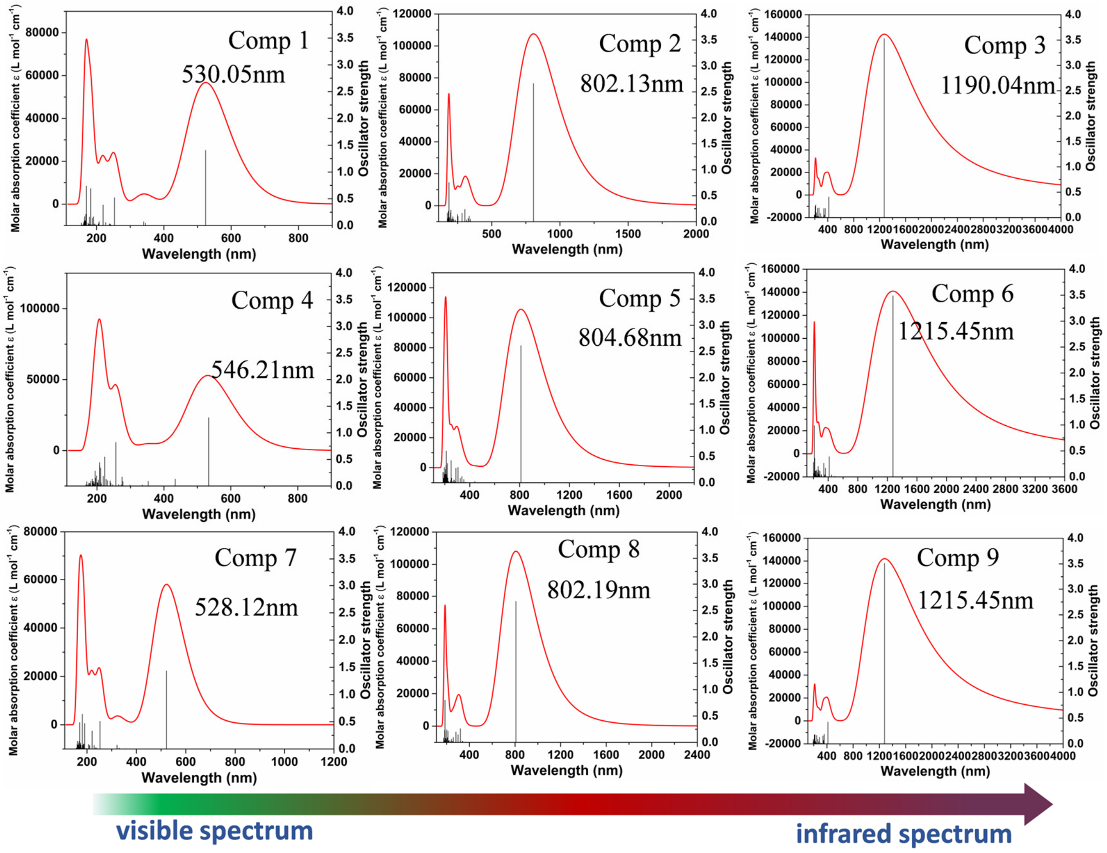


Figure S3. The UV-vis spectra of the nine compounds calculated under the wB97XD/def2-SVP level.
